# Evolution of neuropeptide Y/RFamide-like receptors in nematodes

**DOI:** 10.1016/j.heliyon.2024.e34473

**Published:** 2024-07-15

**Authors:** Franziska Reinhardt, Anette Kaiser, Simone Prömel, Peter F. Stadler

**Affiliations:** a Bioinformatics Group, Institute of Computer Science, Interdisciplinary Center of Bioinformatics, Leipzig University, Härtelstraße 16-18, Leipzig, D-04107, Germany; b Leipzig University, Faculty of Medicine, Department of Anesthesiology and Intensive Care, Liebigstr. 19, Leipzig, D-04103, Germany; c Leipzig University, Faculty of Life Sciences, Institute of Biochemistry, Brüderstraße 34, Leipzig, D-04103, Germany; d Heinrich Heine University Düsseldorf, Universitätsstraße 1/ Gebäude 26.24, Düsseldorf, D-40225, Germany; e Max-Planck-Institute for Mathematics in the Sciences, Inselstrße 22, D-04103 Leipzig, Germany; f Inst. f. Theoretical Chemistry, University of Vienna, Währingerstraße 17, A-1090 Wien, Austria; g Facultad de Ciencias, Universidad National de Colombia, Sede Bogota, Colombia; h Santa Fe Institute, 1399 Hyde Park Rd., Santa Fe, NM 87501, USA

**Keywords:** GPCR, Neuropeptide Y/RFamide-like receptors, Paralogs, ExonMatchSolver, Nematodes

## Abstract

The Neuropeptide Y/RFamide-like receptors belong to the Rhodopsin-like G protein-coupled receptors G protein-coupled receptors (GPCRs) and are involved in functions such as locomotion, feeding and reproduction. With 41 described receptors they form the best-studied group of neuropeptide GPCRs in *Caenorhabditis elegans*. In order to understand the expansion of the Neuropeptide Y/RFamide-like receptor family in nematodes, we started from the sequences of selected receptor paralogs in *C. elegans* as query and surveyed the corresponding orthologous sequences in another 159 representative nematode target genomes. To this end we employed a automated pipeline based on ExonMatchSolver, a tool that solves the paralog-to-contig assignment problem. Utilizing subclass-specific HMMs we were able to detect a total of 1557 Neuropeptide Y/RFamide-like receptor sequences (1100 NPRs, 375 FRPRs and 82 C09F12.3) in the 159 target nematode genomes investigated here. These sequences demonstrate a good conservation of the Neuropeptide Y/RFamide-like receptors across the Nematoda and highlight the diversification of the family in nematode evolution. No other genus shares all Neuropeptide Y/RFamide-like receptors with the genus *Caenorhabditis*. At the same time, we observe large numbers of clade specific duplications and losses of family members across the phylum Nematoda.

## 1 Introduction

Many peptidergic G protein-coupled receptor (GPCR) systems are evolutionarily deeply conserved and are present in all bilaterians [1], [2], [3]. This conservation likely reflects their involvement in fundamental biological functions, such as feeding and reproduction [4], and opens avenues to study their biological regulation in comparably simple protostomian model organisms. Nematodes, in particular *Caenorhabditis elegans* (*C. elegans*), are outstanding model organisms for possibility of genetic manipulation, ease of handling, and translucence. Unsurprisingly, a recent study has suggested that 17 bilaterian-conserved peptidergic systems have at least one ligand-receptor pairing in *C. elegans* [5]. Among them is the neuropeptide Y (NPY) family, which has at least two homologs in *C. elegans*, NPR-11 and NPR-12 [5].

Local gene duplications have led to a large expansion in neuropeptides sharing the C-terminal RF/Yamide sequence signature of NPY. Complementarily, dozens of potential neuropeptide receptor resemblance (NPR) and FMRF-like peptide receptors (FRPR) have been found in this nematode. All of these share significant sequence homology both with NPR-11 and NPR-12 and the human NPY receptors, and display strongly overlapping ligand profiles [5], [6]. Moreover, human NPY Y2 and Y4 receptors are able to functionally substitute NPR-1 in an *in vivo* model of chemo-avoidance, suggesting that there is substantial functional similarity among these receptors [6]. Global phylogenetic analyses in the light of currently available sequence information, however, suggest grouping into three distinct groups: NPR-11 and NPR-12 as “true” NPYR orthologs in the NPY/NPFR bilaterian clade, while sNPF receptors (most NPRs) and FMRFa-like peptide receptors (FRPRs) constitute individual protostomian-specific clades [1], [5].

To better understand the complex relationships and surprisingly similar ligand-binding profiles, we aimed to investigate the phylogenetics of all potential neuropeptide receptors in nematodes, focusing on the following questions:

**(i)** Are there additional neuropeptide receptor-like sequences, in particular in poorly annotated genomes, and (how) does this affect the phylogenetic reconstruction?
**(ii)** Can we identify a minimal set of neuropeptide receptors in evolutionarily ancient nematode groups from which the large repertoires have arisen by local duplication events?
**(iii)** How similar are these most ancient sNPFRs and FRPRs to bilaterian NPFR/NPYR? In particular, are sNPFRs and/or FRPRs co-orthologs with NPFR/NPYRs that have derived from the same ancestor, explaining the strong overlap in biological function?

To tackle these questions, we devised a largely automatized pipeline to search for, retrieve and analyze NPFR and FPFRs. The method systematically utilizes the exon/intron structure of paralogous genes to improve the assignment of genes to paralog groups and to reconstruct gene models from fragmented genome assemblies [7], [8]. This approach enables a comprehensive, detailed analysis of the homologs of several dozens of related *C. elegans* NPFR and FRPR receptors across more than a hundred nematode genomes.

## 2 Methods

**Figure 1:**
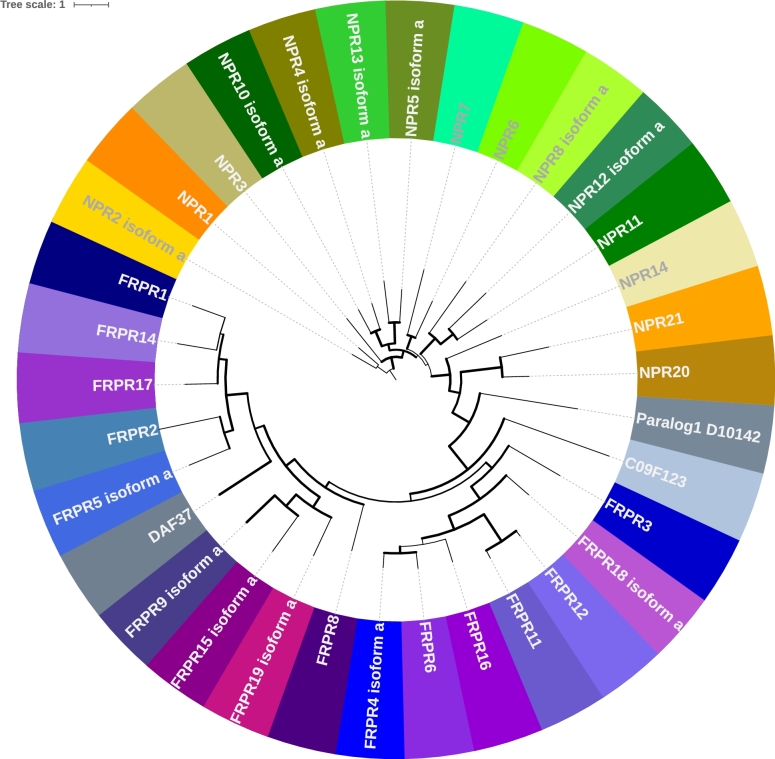
Selected Neuropeptide Y/RFamide-like receptor paralog sequences of *Caenorhabditis elegans* as query. For the classification to NPY/RFamide-like receptors the paper from Frooninckx et al. [9] were used.

Homology searches started from selected Neuropeptide Y/RFamide-like receptor paralog sequences of *Caenorhabditis elegans* (Fig. 1) as query. These query sequences were downloaded from wormbase (https://www.wormbase.org). The splice junctions of the individual exon-intron structure were computed with Exces-A [8]. We downloaded 159 representative nematode target genomes (listed in Supplement 2). We observed that the number of available genomes per genus differs widely (range: 1–22, median: 1, mean: 2.4). Most representative genomes are currently found in the genus *Caenorhabditis* with 22 genomes, followed by *Trichinella* with 12 genomes. The majority of genera (41/66) has only one representative genome.

In order to identify family members and assign them to paralog groups we used the pipeline described detailed in [8]. In brief, it searches for putative homologous protein in the target genomes, uses an exon-centric spliced aligner to determine the intron-exon boundaries and then uses the ExonMatchSolver approach [7] to solve the paralog-to-contig assignment problem. The pipeline was initialized with species closely related to C. elegans (C. japonica, C. nigoni, C. brenneri, C. briggsae, C. bovis). Based on the Hidden Markov Models (HMM) created with the results for each exon per paralog separately, we then iterated the search proceeding from closely related to more distantly related species. After each species, the HMMs were updated and the updated HMMs were used to search in the next species. This yields a collection of alignments of orthologous exons for each sub-family which are used to construct HMMs. These are then used for subclass-specific searches, resulting in a set of high-quality Neuropeptide Y/RFamide-like receptor sequences.

We verified that this procedure recovered previously described NPR and FRPR sequences. Indeed, we found all complete genes annotated. The only data base genes that our search procedure did not identify are incomplete and/or very distant putative homologs.

In order to limit the computational efforts, we subdivided the query sequences into six groups that were processed separately. Group **1** comprises most of the NPRs described [9] (NPR-1, 2 a/b 3, 4, 5 a/b, 6, 7, 8 a/b, 10 a/b, 11, 12, 13) as well as additional paralogs according to wormbase annotation (NPR-9, 18, 22, 27, 31, GNRR2, TKR1, TKR2, TKR3). NPR-14 forms its own group **2**, and NPR 20 and 21 were collected in group **3**. FRPR-1, 2, 3, 4, 5 a/b, 6, 8, 9 a/b, 11, 12, 14, 15, 16, 17, 18 a/b, 19, 20 formed group **4**, while C09F12.3 (group **5**) and D1014.2 (group **6**) were processed separately. These subdivisions were informed by sequence similarity.

ExonMatchSolver assumes that a representative of each paralog group is present in the query set. Moreover, the current implementation faces limitations if the number of paralogs becomes too large. It was not possible, therefore, to process the 187 paralogs (Ensembl 129 paralogs) of FRPR1 in *C. elegans* in fully automatic mode. We therefore limited the input to 45 representative paralogous FRPR sequences and manually curated the output based with the help of gene trees instead of relying on the exon-based HMMs.

Muscle version 3.8.31 [10] were used to generate multiple sequence alignments for the gene Trees, which were computed with IQ-TREE multicore version 1.6.12 with ultrafast bootstrap (UFBoot) [11], [12]. Only resulting sequences with length >100 nt were used for further analysis.

## 3 Results

**Figure 2:**
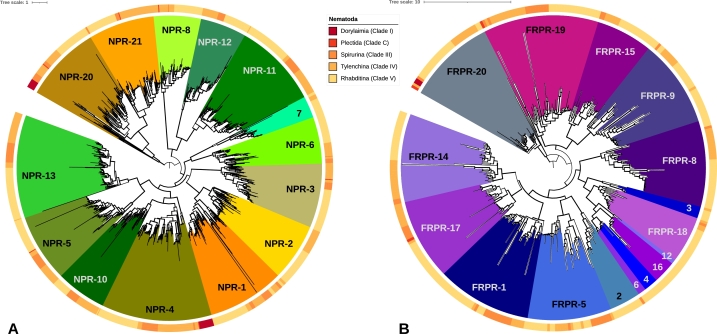
Neuropeptide Y/RFamide-like receptor sequences of Nematodes. A: NPR, B: FRPR. The colors assigned to the receptor families are the same as in Fig. 1.

Starting point for the present analysis were the 41 annotated sequences of NPRs and FRPRs in *C. elegans* reported by Frooninckx et al. [9]. In order to obtain a phylogeny of Neuropeptide Y/RFamide-like receptors in nematodes that is both comprehensive and detailed, we used an automated pipeline [8] to identify and retrieve the homologs of these 41 query sequences in 159 nematode target genomes. Based on the subclass-specific HMMs (Hidden Markov Models, see Methods for details) generated by iterative homology search, we were able to detect high-quality neuropeptide Y/RFamide-like receptor sequences in many species that are currently not well annotated (Fig. 2). Our search suggested three large paralog groups, NPRs (1100 detected and classified paralogs), FRPRs (375) and C09F12.3 (82). The C09F12.3 group is paralog to a GPCR first identified in flies, which had no obvious paralog in previous studies [13]. This is by far the largest and most comprehensive data set of nematode NPRs and FRPRs.

In Dorylaimia, which includes whipworms and Trichinella species, only one of two family members (NPRs and FRPRs) were found, which, with the exception of the single sequence in *Romanomermis*, are classified as NPRs. In contrast, Chromadoria (Plectida, Thylenchina (e.g. *Steinernema*), Spirurina (e.g. *Ascaris*), and Rhabditina (e.g. *Caenorhabditis*)) typically exhibit multiple NPR and FRPR classes, although no FRPRs were found in widely distributed subset of genomes. The most extensive complements are 14 NPRs (in 6 *Caenorhabditis* species), and 11 FRPRs (*Caenorhabditis japonica* and *Parapristionchus giblindavisi*), respectively (Suppl. 1 Fig. 1). On average, each genome harbors 6.3 NPRs and 2.2 FRPRs, not counting near-identical copies.

All four major groups of the Chromadoria have multiple clades of both NPRs and FRPRs. Together with the appearance of at least one NPR or FRPR in the Dorylaimia, this implies that a large part of the diversification of Nematode NPRs and FRPRs took place very early in Nematode evolution, namely in the ancestral lineage of the Chromadoria. Since at present, no suitable representative of the most early branching clade, Enoplia, is available, the exact situation in the Nematode ancestor remains unresolved, although it most likely contained a single NPR and a single FRPR.

**Figure 3:**
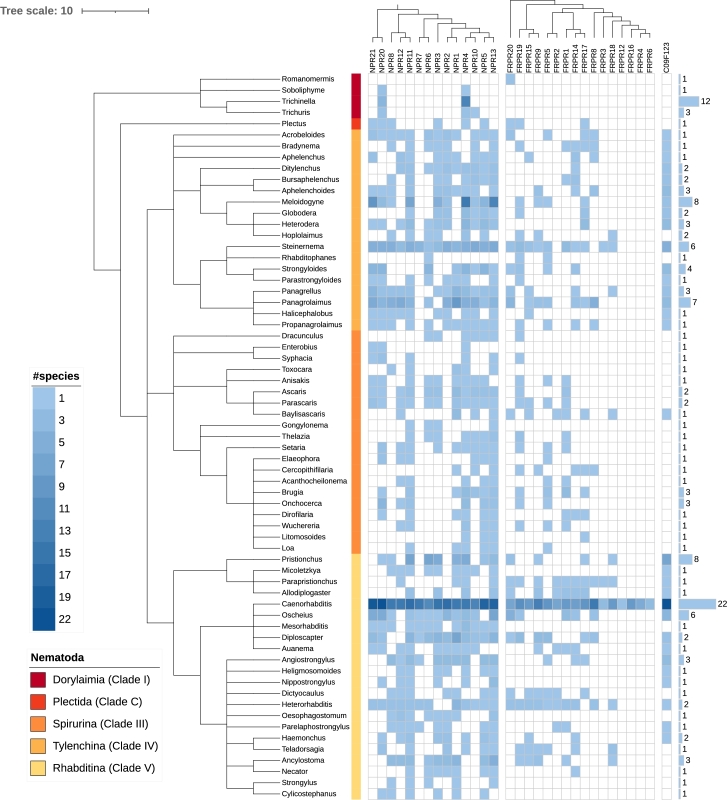
The relationships of the major nematode groups on the left hand side is combined from De Ley and Blaxter [14], Ahmed et al. [15], [16] and Smythe et al. [17]. The gene phylogenies of NPRs and FRPRs on top summarize the results shown in Fig. 2 and Fig. 2. The heat map shown the number of receptors of a given family found in a given genus.

**Figure 4:**
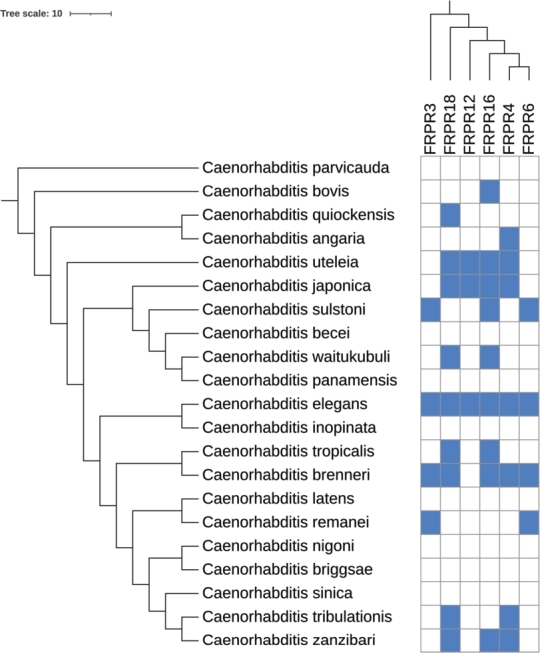
The evolution of FRPR-3, 4, 6, 12, 16 and 18 in the genus *Caenorhabditis* is characterized by frequent, lineage specific losses (light color denotes absence). The phylogeny of the genus *Caenorhabditis* follows Stevens et al. [18], the gene tree of the six FRPRs is extracted from Fig. 2B.

There is also clear evidence for additional, late duplications. For example, NPR-14 and FRPR-11 (not included in Fig. 3) are found in *C. elegans*. A related example of lineage-specific duplications is the exclusive appearance of FRPR-4, -6, -12 and -16 in the genus *Caenorhabditis* only. These genus-specific FRPR paralogs are also subject to frequent lineage-specific loss, (Fig. 4). The receptor D1014.2 is also exclusive to the genus *Caenorhabditis*. No homologs of C09F12.3 are present in Dorylaimia and Plectida, suggesting that this receptor family originated in the ancestor of Thylenchina, Spirurina, and Rhabditina. Since the phylogenetic relationships between these three clades remains contested, it also remains unclear whether these situations have to be explained by duplication or loss events.

Some clades of NPRs are sparsely distributed, suggesting frequent losses. For example, NPR-2 and NPR-7 do not appear in genomes of the Spirurina. Among Thylenchina, NPR-7 was detected only in *Steinernema*. The same can be observed for FRPRs. FRPR-3 was not detected in *Spirurina*, and FRPR-2 is missing in *Thylenchina*.

Additional copies of receptors are also commonly observed. In most cases we found one additional duplicate, and in some cases three paralogous sequences. Copies of NPRs are particularly frequent in the genera *Meloidogyne* and *Diploscapter*. In *Allodiplogaster* additional paralogs of both NPRs and FRPRs were found.

**Figure 5:**
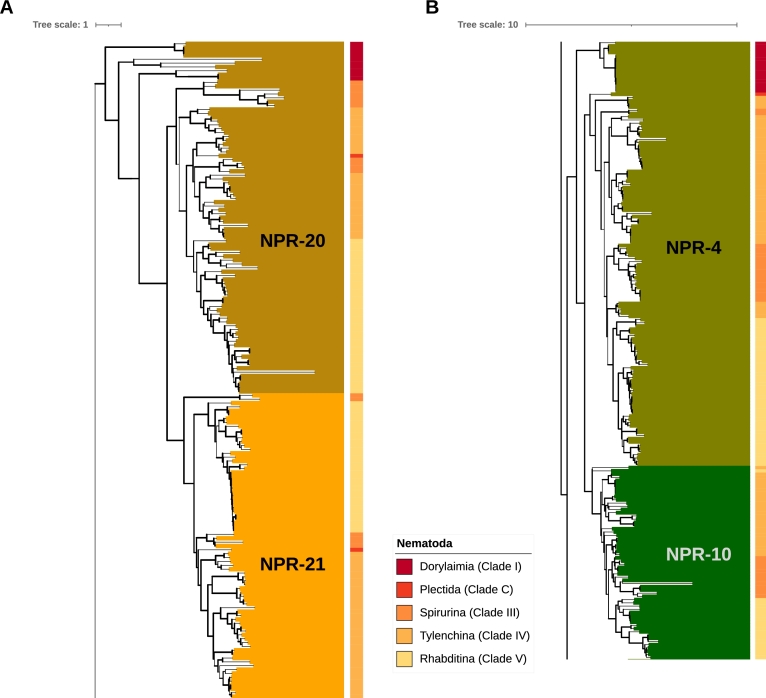
NPR-4 and NPR-20 in Chromadoria. The colors assigned to the receptors in the tree corresponds to Fig. 1. The genes identified as NPR-4 and NPR-20 in Dorylaimia should be regarded as direct descendants of the common ancestor of NPR-4 and NPR-10 as well as NPR-20 and NPR-21. A: NPR-20 and NPR-21 B: NPR-4 and NPR-10.

The genes identified as NPR-4 and NPR-20 in Dorylaimia should be regarded as direct descendants of the common ancestor of NPR-4 and NPR-10 as well as NPR-20 and NPR-21. These two groups only diverged in Chromadoria, see Fig. 5 (A, B).

*Characteristic sequence motifs*

**Figure 6:**
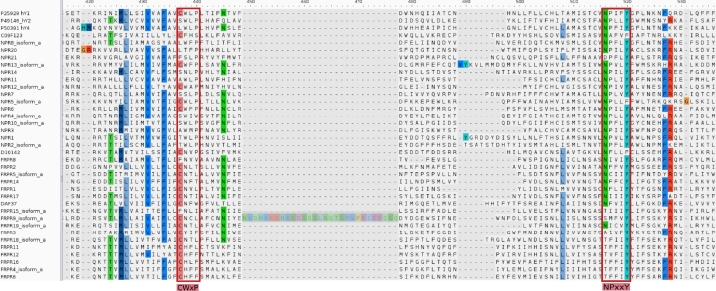
Alignment of different *C. elegans* NPRs and FRPRs in comparison with human NPY receptors. The Alignment shows the motif CWxP in TM6 and the NPxxY motif in TM7.

NPRs and FRPRs differ substantially in terms of conserved sequence motifs. While NPRs harbor all typical sequence signatures of rhodopsin-like GPCRs, this is not the case for nematode FRPRs. This is readily seen in an alignment of *C. elegans* NPRs with human NPY receptors, see Fig. 6.

NPRs contain most of the well-known microswitch residues of Rhodospin-like receptors in TM6 (CWxP) and TM7 (NPxxY), although there are small variations in individual NPRs. For example, NPR-4 does not have the typical motif CWxP in TM6, but S/CWxW; in NPR-11 it is AWLP. The NPxxY motif in TM7, however, is broadly conserved. NPRs and FRPRs clearly belong to different gene families, although both can be grouped together as potential NPY-like receptors: FRPRs lack (a) typical and functionally essential sequence signatures of NPY-like receptors such as D/E6.59, E45.52, and Q3.32 [6], [19], and (b) display great variation from typical rhodopsin-like signatures (DRY/ERY in TM3, CWxP/SWxP in TM6, NPxxY in TM7 [20]). In FRPR, the NPxxY motif in TM7 is significantly altered and lacks the typical proline, and frequently the asparagine is replaced by threonine. Interestingly, however, the tyrosine is also conserved. The conserved Tyr is often followed by another Phe/Tyr. Interestingly, neither the Trp toggle switch nor the kink-inducing P of the CWXP motif in TM6 are conserved in FRPRs. Instead, the W is replaced by hydrophilic H/N/Y. In contrast, in NPRs the CWxP motif is visible in most cases and W and P are well conserved.

Moreover, FRPRs do not appear to have an extracellular disulfide bond between TM3 and ECL2. Instead, the folding of ECL2 occurs by hydrophobic packing with an extracellularly extended TM3 according to AlphaFold2 models of *C. elegans* FRPRs [21], [22]. This suggests a third condition of typical sequence motifs of Rhodopsin-like GPCRs is violated and poses questions how these receptors function. Among the nematode NPRs, finally, NPR-20 has a hybrid structure. The CWxP motif in TM6 is incomplete and only shows the typical proline, while the NPxxY motif in TM7 is conserved and two cysteines would allow the formation of a disulfide bridge between TM3 and ECL2.

## 4 Discussion

Here, we report a systematic survey of Neuropeptide Y/RFamide-like receptors across 160 Nematode species (*C. elegans* as query + 159 target genomes). The density of our data set is sufficient to study gene turn-over, i.e., clade-specific gains and losses in detail. We found that nematodes have acquired an extensive, clade-specific repertoire of these receptors starting from a very simple ancestral state. The expansion of the Neuropeptide Y/RFamide-like receptors through a multitude of subgroup-specific duplications was complemented by extensive, independent gene losses, in particular among FRPRs. Our survey is not the first systematic study of this receptor family. It provides, however, by far the most extensive data set analyzed to date. In addition to drawing comprehensive global picture of the complex evolutionary history of this gene family, our data at least in part confirm many of the observations from previous, smaller, studies.

McCoy et al. [23] reported that NPR-7, FRPR-4, FRPR-6, FRPR-11, FRPR-12 and FRPR-16 have evolved by gene duplications within the genus *Caenorhabditis*. Our results are partly consistent with this report. We indeed found FRPR-4, 6, 12 and 16 only in *Caenorhabditis* (Fig. 3), confirming the observations by McCoy et al. [23]. The receptor FRPR-11 was only found in the query *C.elegans*, indicating a very recent duplication. In contrast to earlier reports, we also found NPR-7 orthologs in *Steinernema* in the *Thylenchina* group, suggesting an earlier duplication. Such differences are not surprising because McCoy [23] considered only 17 species while the present study is based on a much denser taxon sampling covering 159 species. Furthermore, we used an iterated homology search procedure that is designed to ensure that gene families are included as completely as possible.

Cardoso et al. [24], using only the sequences of Neuropeptide Y/RFamide-like receptors in *C. elegans*, suggested that the pairs {NPR-4,NPR-10}, {NPR-5,NPR-13}, and {NPR-11,NPR-12} are likely the result of recent duplication. Our much larger data set confirms this hypothesis. In addition, we find that {NPR-20,NPR-21} and {NPR-6,NPR-7} are the results of recent gene duplications, see Fig. 3.

Atkinson et al. [25] analyzed 15 neuropeptide GPCRs in 10 nematode species and reported that there are fewer neuropeptides in the Dorylaimia group than in the other clades. This observation matches our data. On the other hand, there are major discrepancies in the number of GPCRs present between *Trichinella/Trichuris* and *Romanomermis*. Our data suggest that there are fewer losses in the *Trichinella/Trichuris* clade. Instead many of the paralog groups absent in Dorylaimia are much more like the result of duplications that occurred later within the Chromadorea. The presence of 6 of the 14 NPR groups in *Plectida* serves as additional support for this scenario. In Dorylaimia, NPR-20 and NPR-4 were found in three of four genera. Our data suggest that a single (or at most two) NPRs were present in the ancestor of Dorylaimia. Regarding FRPRs, only a single representative (FRPR20 in *Romanomermis*) was observed in Dorylaimia. The most likely scenario therefore is either a single or possibly two NPR gene(s) and a single FRPR in the nematode ancestor, from which the extensive diversity of receptors in Nematoda originated entirely within the Nematoda.

We also confirm the observation that *Ascaris* (Spirurina) has a larger number (13/31) of neuropeptide GPCRs than *Haemonchus* (9/31) and *Necator* (8/31) (Rhabditina), even though the latter are more closely related to *C. elegans*, which served as source for the query sequences. This suggests more extensive losses in many of the Rhabditina genera.

The expansion of protein families in nematodes in general was demonstrated by means of cluster analysis [26]. For *Dorylaimia* (*T. spiralis*, *T. muris*, *S. baturini*) however, a reduced set of GPCRs was reported. As an exception, *R. culcivorax*, has a more standardized set of GPCRs. The overall gene content shows a similar pattern. This is also reflected in the differences in gene content of this group. Interestingly, the NPR and FRPR in *R. culcivorax* match the other *Dorylaimia*. The number of GPCRs generally increases during evolution. This is particularly striking in the group of chemosensory class A receptors. Here, we observe a similar behavior for neuropeptide GPCRs in nematodes.

Additional copies of receptors are also commonly observed in our study. For example, a recent study demonstrated whole genome duplication (WGD) in *Allodiplogaster sudhausi* [27]. All 4 NPRs and 4 of 6 FRPRs we found have an additional copy. Other known WGDs in *M. incognita* (triploid) [28], [29], *H. glycines* (tetraploid) [30], and the panagrolaimid nematodes [31] are also reflected in the doubling or even tripling of the number of single paralogous sequences.

Surprisingly, nematode FRPRs exhibit some highly unusual sequence features that underline their distinct evolutionary origin. Although both NPRs and FRPR belong to Rhodopsin-like GPCRs, nematode FRPR lack some of the most characteristic sequence features of this receptor family that are involved in relaying receptor activation. With distinct alterations in the CwxP (TM6) and NPxxY (TM7) motifs, it remains enigmatic how ligand binding is communicated to effector proteins inside the cells in the absence of these microswitches. Nonetheless, a recent large-scale functional analysis of the NPR peptidergic system showed activation of a promiscuous G16 protein by some FRPRs [5], suggesting alternative microswitches to promote G protein activation. In addition, sequence patterns in the extracellular parts of the receptors that are specific for NPY-like receptors such as D/E6.59 and E45.52 (ECL2) are missing in FRPRs, making it tempting to speculate that the molecular pattern of ligand binding is distinct from NPRs [19], despite the conserved RF-amide sequence of the FLP-ligands. Biochemical studies on the functionality of FRPRs in contrast to NPRs will be necessary to investigate the functional impact of these changes. For several *C. elegans* NPRs, a coupling to different human G proteins has been shown (e.g., [6], [19], [32], while the knowledge about function and molecular mechanisms of receptor coupling of the nematode arrestin homolog have so far remained scarce across the entire GPCR family [33], [34].

For instance, direct coupling of FRPRs to G proteins or Arrestin could be investigated to confirm their mechanism of action, as the CWxP and NPxxY motifs are well-known microswitch regions related to receptor activation.

The expansion of NPRs/FRPRs in the Nematodes and the resulting diversity suggest that the system is functionally highly essential. Together with the multitude of neuropeptides that can bind to the receptors, it provides a large repertoire of peptide-receptor interactions in the nervous system [5]. Its very defined and relatively simple nervous system makes *C. elegans* (among other features) a great model organism [32] in the field of neuroscience. The large neuropeptide receptor system allows for precise, orchestrated, and context-specific signaling between neurons and thus, complex regulations. Studies have shown an involvement of neuropeptide signaling in various processes such as for instance in feeding and social behavior [35], [36], [37], [38], the response to ethanol [39], and thermotolerance [40]. The data compiled in our study can in the future be used to further shed light on the functions of the neuropeptide systems. The study puts a spotlight on the surprisingly distinct origin and sequence characteristics of NPR and FRPR receptors, respectively. Based on our results, more in-depth functional characterization of FRPR receptors is required in order to dissect the complex neuropeptidergic system in nematodes.

## 5 Concluding remarks

Nematodes have evolved an extensive system of neuropeptide Y/RFamide-like receptors starting from an ancestral state that most likely comprised a single NPR and a single FRPR gene. The evolution of this gene family was characterized by both, many clade-specific duplications, and an extensive parallel gene losses. In general, NPRs seem to be better conserved than FRPRs, where clade-specific losses appear to have been particularly abundant. This complex pattern also suggests prolific functional innovation as the most parsimonious explanation for the many new paralog groups, in particular among the FRPRs. Even though we have investigated 160 genomes (*C. elegans* as query + 159 target genomes) in considerable detail, many of the details of gains and losses remain a topic for future research. The reason is that the quality of most nematode genome assemblies are insufficient to firmly conclude that a paralog that was not observed is truly absent. It is thus impossible to conclusively distinguish gene losses from missing data. Nevertheless, our data are sufficient to place the origin of most paralog groups (with the notable exception of NRPR-4, -6, -12, -16) already early in the Chromadorea. In addition to the insufficient quality of some genomes with regard to missing parts, there is also the possibility of incorrect genome duplications, which can lead to incorrect extensions of genes independent of the known genome duplications.

An even more fine-grained analysis of the evolution of Neuropeptide Y/RFamide-like receptors will require an even more dense taxon sampling, and in particular a much better coverage of the currently underrepresented (Plectida, Dorylaimia) and missing (Enoplea) clades.

## Abbreviations

C. elegans: *Caenorhabditis elegans*
**FRPR**: FMRF-like peptide receptor
**GPCR**: G protein-coupled receptor
**HMM**: Hidden Markov Model
**NPR**: neuropeptide receptor
**NPY**: neuropeptide Y

## CRediT authorship contribution statement

**Franziska Reinhardt:** Writing – original draft. **Anette Kaiser:** Writing – original draft. **Simone Prömel:** Writing – original draft. **Peter F. Stadler:** Writing – original draft, Funding acquisition.

## Declaration of Competing Interest

The authors declare that they have no known competing financial interests or personal relationships that could have appeared to influence the work reported in this paper.

## Data Availability

A multi-fasta file of the Neuropeptide Y/RFamide-like receptor sequences is available as Supplemental Material.

## Appendix A Supplementary material

MMC 1 Evolution of 30 Neuropeptide Y/RFamide-like receptors in Nematodes. MMC 1

MMC 2 Nematode species (including Assembly, BioProject ID, Clade, N 50, Genome Size). MMC 2

MMC 3 Resulting Multiple Sequence Alignment. MMC 3
