# Supplementary material for: Evolution of neuropeptide Y/RFamide-like receptors in nematodes
Source: Heliyon. 2024 Jul 15;10(14):e34473. doi: 10.1016/j.heliyon.2024.e34473 (PMC11315170; doi:10.1016/j.heliyon.2024.e34473)
Supplement: MMC 1 — Evolution of 30 Neuropeptide Y/RFamide-like receptors in Nematodes. [file mmc1.pdf]

1 Supplemental Material

References

M. Ahmed and O. Holovachov. Twenty years after de ley and blaxter—how far did we progress in understanding the phylogeny of the phylum nematoda? *Animals*, 11(12):3479, 2021.

M. Ahmed, N. G. Roberts, F. Adediran, A. B. Smythe, K. M. Kocot, and O. Holovachov. Phylogenomic analysis of the phylum nematoda: conflicts and congruences with morphology, 18s rrna, and mitogenomes. *Frontiers in Ecology and Evolution*, 9:769565, 2022.

P. De Ley and M. Blaxter. Systematic position and phylogeny. In *The biology of nematodes*, pages 1–30. CRC Press, 2002. doi: 10.1201/b12614-2.

A. B. Smythe, O. Holovachov, and K. M. Kocot. Improved phylogenomic sampling of free-living nematodes enhances resolution of higher-level nematode phylogeny. *BMC Evolutionary Biology*, 19:1–15, 2019.

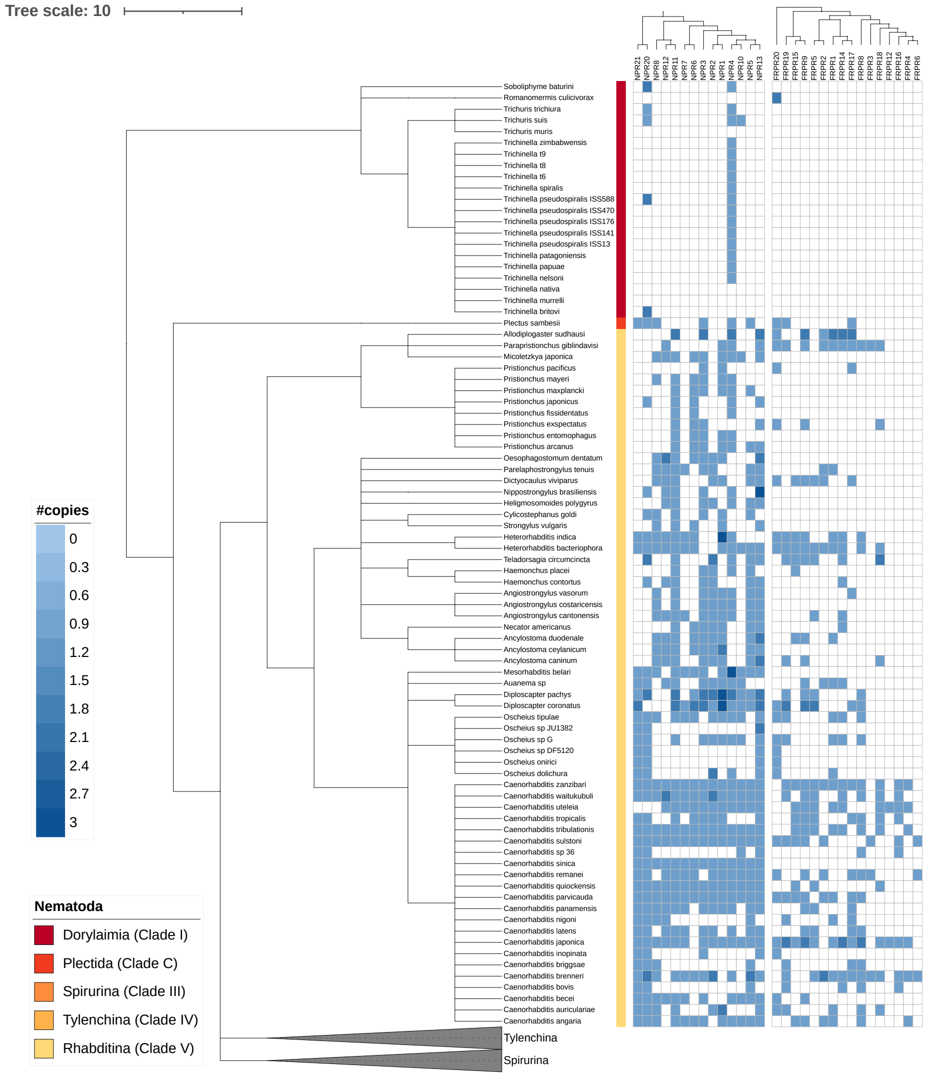

Figure 1: *Evolution of 30 Neuropeptide Y/RFamide-like receptors in Nematodes. The relationships of the major nematode groups on the left hand side is combined from De Ley and Blaxter De Ley and Blaxter [2002], Ahmed et al. Ahmed and Holovachov [2021], Ahmed et al. [2022] and Smythe et al. Smythe et al. [2019]. The phylogenies of the NPRs and FRPRs are taken from the results (Fig. 1). The figure shows how often the specific receptor could be found in the species.*

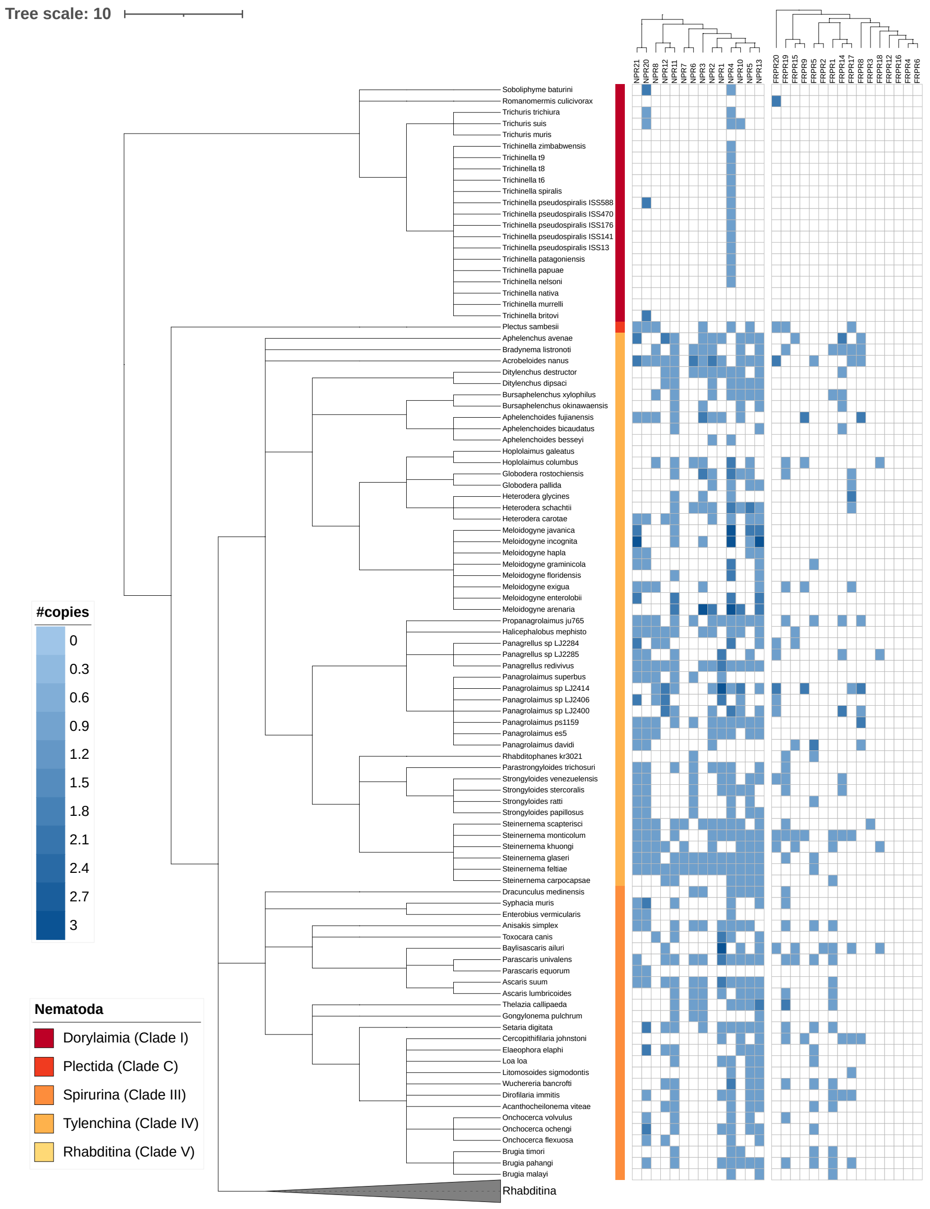

Figure 2: *Evolution of 30 Neuropeptide Y/RFamide-like receptors in Nematodes. The relationships of the major nematode groups on the left hand side is combined from De Ley and Blaxter De Ley and Blaxter [2002], Ahmed et al. Ahmed and Holovachov [2021], Ahmed et al. [2022] and Smythe et al. Smythe et al. [2019]. The phylogenies of the NPRs and FRPRs are taken from the results (Fig. 1). The figure shows how often the specific receptor could be found in the species.*
